# Dysenteria Intermittent

**Published:** 1856-07

**Authors:** Henry W. Davis

**Affiliations:** Paris, Ill.


THE NORTH-WESTERN

MEDICAL AND SURGICAL JOURNAL.

(NEW SERIES.)

VOL. V. JULY, 1856. NO. 7.

### ORIGINAL COMMUNICATIONS.

ARTICLE I.—*Dysentaria Intermittent.* By HENRY W. DAVIS, M.D., Paris, Ill.

During the past summer and autumnal months, various localities in the eastern portion of our state were visited by an epidemic of flux, which, in all places, was of rather an aggravated character, and in some it assumed a very virulent type. In no place that I could hear of were the attacks of this disease characterized by so many alarming symptoms at the outset, nor did they present so many phases difficult to control, as peculiarized those cases which occurred in the town of Paris and its vicinity. It is of this epidemic, as it fell under my observation, that I wish to say a few words, confining myself exclusively to its pathology and therapeutics. I will not attempt anything like a topographical history of the disease, as it had no defined limits, and appeared to affiliate with none of the tangible causes, which are too often readily found and easily recognized as the promoters, if not the originators, of poisons productive of fatal diseases.

It is true it orignated among persons who were surrounded by every auxiliary calculated to invite an attack of the disease in any shape, to render it more virulent in character, and predispose it to a fatal termination; while, from the length of time which it remained confined to one spot, hopes were entertained that it would be limited to the household among whom it first made its appearance. But its gradual spread disabused our minds of the idea that it might have been the result of some local cause, and convinced us that the origin of the disease was not only general in its character, but that its influence would be pretty generally felt and manifested. Under all circumstances, and in all places, it made its appearance; filth seemingly offered no attractions, nor added to its virulence—cleanliness presented no safeguard, nor abated one jot of its severity.

Manifesting these characteristics, it was not strange that we should look among the many morbific causes engendered in the climate, and which are peculiar to the sloughs and swamps of the Wabash Valley; for the controling influence, if not the specific cause of the disease—and I think the evidence of the operation of at least one of them, was so unequivocal as not to leave the slightest doubt on the mind of any one who sought the sources and examined the workings of the disorder.

Having myself started but a short time since in the practice of medicine, and selecting a field of operation in the Valley of the Wabash, I was, to a great extent, unprepared to recognize the almost unlimited control which the malaria of this region exerts on many diseases which, to a casual observer, are apparently extraneous to its influence. Diseases which have hitherto been regarded as idiopathic in their character, arising from certain specified causes, running a peculiarly independent course, and controlled by remedies which seemingly had no immediate bearing upon, or connection with, the antagonistic agents of malaria, are found to be the veritable offspring of the great father of western diseases. I do not wish to be understood as asserting that diseases of a similar nature may not arise when the specific poison does not exist; but when they do fall within the sphere of its influence, they maintain all the essential characteristics pertaining to diseases of a strictly malarious origin: the same class of symptons is recognized, and the same remedies are indicated for their subjection.

Of all the diseases thus brought within the control of miasmatic poison, there is not one in which the effects of said poison are more peculiarly manifested than in the one under consideration. It is a disease which may and does prevail in localities where the malaria of intermittents is unknown; but that does not militate against the fact that, as the disease prevails among us during the summer and autumnal months, it not only appears to be completely under the sway of miasmatic poison, but it may be said to be the direct effect of it.

I believe that there is more malarious influence exerted in this disease, even in sporadic cases, and that it is to a greater extent under its specific control, than any other which may arise independent of the poison. And when the disease is in the form of an epidemic prevailing over a large extent of country, and where the most marked symptons are manifested in almost every case, all indicating the one and only morbific cause, I think I hazard little in saying that dysentery, in its epidemic form, as it obtained with us during the past summer, is just as much a consequence of the malaria generated and evolved in this region, as is ague or any of its kindred complaints.

It is universally admitted that when there exists any general morbific cause, unless it involves some specific poison, nature, after presenting every obstacle to its encroachments, will succumb in that portion of the human organism previously debilitated, or upon which the causes of disease most readily exert their influence; and thus results a variety of diseases from a similar cause. In our climate, surcharged as the atmosphere is with morbific influences, there must be one or more of the organs essential to life which will be subjected to their control; and there will be generally an attending derangement of the organ or organs, in the great majority of cases, which will require especial attention and treatment. So universally is this the case, that we are always on the alert to ascertain the condition of the suspected part prior to forming an opinion in any disease.

The liver, by far the largest and most important of all the secerning organs, is the one upon which the impress of malarious poison is first felt and soonest manifested; and to it, in a deranged condition, do I look for the fountain and origin of the disease called flux. To be more explicit, I will say that I regard the irritation, inflammation, and congestion of the mucous membrane of the intestine, *sequelæ* of an existing disorder of the biliary organ, and that disorder to be the result of malarious poison acting upon it.

The first result, at least we will assume it to be such, of the impress of the poison is congestion; and admitting that there are no other organic derangements, we think that alone is sufficient to produce all the sequents which are attendant upon an attack of flux. When this congestion obtains, the primary result is deficient secretion of bilious matter, or the production of a vitiated article unfit to serve the purposes for which nature designed it; or providing that the secretion of bile is not suspended, its elimination is obstructed to a degree that renders its passage into the intestine a thing impossible. Hence we have an entire absence of bilious discharges in dysentery; and whether we look upon it as obstructed elimination or morbid secretion, it is in either case calculated to bring on an irritable condition of the mucous membrane of the intestine, and which may be regarded as the commencement of the disease in that part. The second consequence of the hepatic congestion, is the mechanical obstruction which is presented to the return of the blood through the natural channels to the heart; the equilibrum of the circulation is destroyed, and consequently it is thrown back, or kept back, in the veins, and held as it were inert. The mesenteric veins receive their share of the suspended blood; and, in turn, the capillary net work, which is spread over the mucous tissues of the large intestine, becomes surcharged with an undue quantity.

Here, then, we have irritation of the intestines, from the altered or suspended secretion of bile, with that amount of congestion which is always attendant upon irritation, and we also have the congestion which is the result of the engorgement of the portal circle. The sequents of this condition of things are, as I conceive, the following: altered secretion from the surface of the mucous membrane from the want of proper stimulus in the shape of healthy bile, and from the congestion which prevents the normal action of the secreting vessels; rupture of the capillaries from over distension, and an effort on the part of nature to relieve the congestion; and inflammation which results from overdistension and rupture—blood and mucous thus find their way into the intestines, and we have the discharges which are characteristic of dysentery.

This, in a few words, is an outline of what I believe to be the true pathology of flux. It might be carried still further through all its sympathetic derangements, whereby other organs become involved, and the whole organism made subservient to the disease; but neither this, nor any of the untoward symptons which may indicate these extraneous lesions, will mar or in any degree interfere with the pathological account of the disease, as I have here attempted to write it. It is no new theory, so far as its malarious origin is maintained; and yet those who have endorsed it heretofore have, it seems, signally failed in applying those remedies which a rational accordance with this pathological view would suggest, and we can scarcely hope to arrive at any successful plan of treatment, until our views with regard to the pathology of the disease are well settled upon a reasonable basis, and the remedy selected in unison with it.

Now, the question arises, what shall the agent or agents be which such a morbid condition of the system demands for relief? I believe there is not a practitioner of medicine who has not at times felt the inefficiency of the means recommended by the ablest authority, and which belong to the usual routine of treatment when called upon to prescribe for the disease. As for myself, although having but little experience in the complaint as a medical adviser, I never felt any confidence or satisfaction in conducting a case of dysentery either after the ordinary mode as prescribed in the text-books, or the plan pursued in hospital practice in the East—as it was always impossible for me to say with any certainty whether my patient would be better or worse during the coming twenty-four hours. I gave the usual remedies without much faith in their power to do good, and left the case in the hands of Providence until the next visit. Among these remedies the most prominent were mercurials, opiates, mineral and vegetable astringents, oleaginous and terebinthinate preparations. *With* them, or *in spite* of them, most of the patients got well; yet in many cases it was questionable whether the disease or the patient would come off the best. They certainly did not work satisfactorily; and I was ready and willing to adopt any other mode of practice which bore the appearance of rationality and promised a better success. Holding, as I did, the pathological views I have here attempted to recite, and believing them to be true, I could not consistently follow the old track, but was compelled to discard the former method of cure, and adopt something in accordance with these views, mercury was no longer admissible, because it failed to meet the end for which its exhibition was designed. If any good resulted, it was temporary, and was more than counterbalanced by the irritation it produced in the already irritated organs; and it in no wise contributed to produce that condition of things which must precede a cure, and through which it can alone be effected. It is true it produced its specific effect on the liver, and one or two bilious discharges were the result; but this had but a small tendency to cure the disease. It relieved little or none of the existing congestion, and added to, rather than allayed, the irritation; and I have no faith whatever in the doctrine which teaches that the passage of bilious matter over the inflamed surface of the intestine has a tendency to restore it to a normal condition. So far from it, I believe it has a diametrically opposite effect. Every unhealthy secretion eliminated from the secerning organs is an irritant; and the matter evolved from the liver, in its abnormal condition, instead of acting as a curative, only adds fuel to the flame.

Astringents, which have always been regarded as powerful adjuvants to a mercurial practice, and upon which many place their whole reliance, had to be discarded, because they had a direct tendency to counteract the efforts which the *Vis Medicatrix Naturæ* might make to produce relief; and also to militate against the good effects which proper remedies might engender. If the pathology here laid down is correct, not one good and sufficient reason can be given to justify their employment. If they act topically, they increase the amount of irritation, and operate against the only effort which nature can make to relieve the congestion of the mucous membrane of the intestine. If taken into the circulation by absorption, their force is spent upon the arteries, producing a reduction of their calibre, and, of necessity, forcing upon the already distended veins the surplus blood. And, moreover, it seems to be bad policy to give any remedy which has a tendency to retain within the intestine the effete matter which is thrown off as the debris of the disease; so that of all the remedies which had been previously used by me, I should feel myself authorized in employing but one, and that is opium.

Before speaking of the remedial agents, (which, in my hands, seemed to have a happy and almost specific effect in removing the morbid condition of the affected organs, and a tendency to coincide with the efforts of nature to restore them to a normal condition,) I wish to say a few words in relation to what I believe to be the leading object to be attained in the treatment of flux, and without which no immediate or permanent amendment can take place.

Having endeavored to show that the whole train of symptoms connected with the disease is a consequence of a deranged condition of the liver, and that derangement involving congestion as the leading sequent of the morbific effects of malaria: the primary object would be the relief of the organ from engorgement, and the restoration of the balance of circulation; as success, in accomplishing this, would be as certainly followed by a removal of the enteric symptoms. When the liver pours its contents into the intestines, the mechanical obstruction to the return of the blood to the heart is removed; the circulation becomes equalized, and it is no longer forced back into the abdominal veins, and thence through their ultimate ramifications, which constitute the capillary net-work of the mucous tissue. Nature is no longer powerless in the disease, but can exert all the resources of her economy to assist in bringing about convalescence. The cause of the disease is virtually removed; and, simultaneous with the disgorgement of the portal circle, we have a cessation of the bloody mucus discharges, release from the tenesmus and tormina, and the substitution of bilious alvine dejections, which, when continuous, are a certain indication of returning health. It may be, that some of you will detect in the foregoing sketch of the pathology of dysentery, many imperfections and discrepancies which my experience has caused me to overlook; yet it does seem to me, that there is something reasonable in it: and when we follow the disease from its rise to its termination, and consider, in connection with it, the evidences of its decline, the whole seems to work together very nicely.

In speaking of the treatment of the disease, little need be said, as it is simple in its character, although not more so than it is effective and satisfactory in its results. It has been highly recommended, and, in the hands of some practitioners, eminently successful. It is not a new idea, as it was employed and its use advocated many years ago, although I had nowhere seen any account which attempted to put the practice in connection with the pathology of the disease, until I met with an essay from the pen of Dr. Read, of Terre Haute. He is the first writer I have read after, who explained the *modus operandi* of the saline treatment, and placed it upon a strict physiolgical and pathological basis; and, although I differ with him as to the ways and means through which the remedy produces its specific effects, still, the reasons adduced by him are sufficiently strong to warrant its employment; whilst the peculiar ideas which others may entertain, in nowise operate against, but serve to strengthen, the rationality of the practice.

Sulphate of magnesia, or epsom salts, is the remedy which I used in the treatment of flux during the past summer; and with it, I have not only had the satisfaction of seeing my patients recover, but I felt a confidence in it which I derived from the use of no other remedy; and, I must say, that it comes nearer a specific than any medicine ever employed by me. I tested it fully and fairly; and in but few instances did it fail to endorse the praises which had been bestowed upon it. In many cases I trusted to it alone, and, with but few exceptions, it succeeded in bringing about the great desideratum: that is, producing and maintaining free and copious bilious discharges. In some instances, where the symptoms were of a serious character, I employed other remedies as adjuvants to a tentative treatment; but this was the remedy upon which I most relied, and which I never failed to use. With regard to its operation, I will say, that I view its effects as being altogether topical; that it produces relief by its action upon the parts secondarily affected in the disease; and that it is a depletive and a tonic.

When the salts are administered, their first operation, after leaving the stomach, is upon the duodenum, which is the primary intestinal receptacle of the bilious matter, and into which the ductis communis opens. The closure of the mouth of this duct, I regard as one of the sequents of the hepatic derangement, and one of the main obstacles to the passage of the bilious secretions into the intestine. This obstruction of the duct is occasioned by the congestion which is consequent upon irritation; for *ubi irritatio ubi fluxas*, is a truism which is applicable to all parts of the human organism. The saline mixture here produces its first impression towards the removal of the disease or its causes: it operates upon the mucous surface of the intestine where the duct terminates, and, by depleting the congested capillaries, it not only removes the mechanical obstruction to the passage of the bile, but it invites its flow through its proper channels.

But it is not here alone that its good effects are manifested, for, during its passage through the primæ viæ, as it passes over the inflamed and congested surface of the intestines, its peculiar operations are produced here as elsewhere. Copious serous discharges are produced; the engorged vessels are relieved; and nature, no longer overtasked, can exert herself to assist in removing the superabundant blood. The secretory vessels cease to be inactive, and resume their labors, and thus the medicine comes to be regarded as a tonic.

The two great objects in the treatment of flux, are in this way reached: First, the relief of the engorgement of the liver; and, secondly, the equalization of the circulation. The mode of preparing the salts, is to make a saturated solution in mint water, adding sufficient comp. spts. lavender to color it nicely and impart a pleasant flavor. The dose is one tea-spoonful every two hours.

The other agents used by me, in connection with the salts, were opium and quinine; both of them were essentially necessary in some cases, while the latter is imperatively demanded in almost all. With regard to the employment of the first, I must say, that in this disease, I accord to it more than the properties of a sedative. I used it in every case where there was any considerable amount of pain, or great annoyance from frequent calls to the chamber; and gave it in large doses, (say from 1 to 2 grs., according to the urgency of the symptoms,) and repeated it every two or three hours, until relief was obtained. I usually exhibited it at night; always regarding the rest and freedom from pain which the patient might enjoy from its influence, as essentially necessary to husband the strength, and aid the *visrecuperandis;* and when, after using the salts from ten to twelve consecutive hours, bilious discharges were not produced, one or two large doses of opium seldom failed to be followed by the desired result. The effects of the opium are, I judge, manifested in the relief of the muscular contraction which is attendant upon irritation. Not only the mouth of the ductus communis, but the smallest capillaries are in a state of tonic contraction from this cause. The opium, in large doses, has a tendency to relieve this, and materially to assist in removing the mechanical obstructions to the free exit of bile and the regularity of the circulation.

Quinine is the last, but not the least, important of the remedies which I think are indicated by the symptomatology of flux. It is eminently essential in preventing the organic congestion, after relief is obtained by the use of other remedies; and its administration is strongly indicated by the fact, that in no serious case of flux, where the symptoms were sufficiently aggravated to require close attention and observation, did I fail to detect a periodical exacerbation. I did not expect it or recognize it by a chill, or the febrile symptoms consequent upon it; but if, during the twenty-four hours, I was enabled to ascertain that, at any particular period, there were manifest any evidences, either extraneous to the disease or immediately connected with it, I regarded it as sufficient indication for the use of quinine, and administered it accordingly.

Often there were no other manifestations than an increase of pain, attended with more frequent calls to the chamber. Sometimes it was denoted by difficult breathing; but, let the indication be what it would, if it retained periodicity as one of its characteristics, I did not hesitate to use quinine, and that, too, in very decisive doses.

If I have succeeded in what I designed, I have shown how flux is a consequence of the operation of malaria upon the liver; and in what way the sulphate of magnesia operates in relieving the morbid condition of the afflicted parts; how opium acts, not only as a palliative, but as an effective adjuvant to the saline treatment; and the necessity which the symptoms indicate for the use of quinine to obviate the return of the congestion. I may have enlarged upon some points that were of little interest, and left untouched those that merited particular notice; but it would have been adding prolixity to what is already too long, to have attempted a more extensive view of the subject, while it would be impossible to carry an essay much further without trying the patience beyond reasonable limits.

